# Molecular Characterization of *Klebsiella pneumoniae* Clinical Isolates with Elevated Resistance to Carbapenems

**DOI:** 10.2174/1874285801711010152

**Published:** 2017-07-31

**Authors:** Rasha Barwa, Mona Shaaban

**Affiliations:** Department of Microbiology and Immunology, Faculty of Pharmacy, Mansoura University, 35516, Mansoura, Egypt

**Keywords:** Carbapenem resistant, *Klebsiella pneumoniae*, Copy number of carbapenemases, Outer membrane proteins, RAPD

## Abstract

**Background:**

Emergence of carbapenems-resistant *K. pneumoniae* represents a serious challenge for antimicrobial therapy.

**Objective:**

The aim of this research is to determine different mechanisms mediating the emergence of *K. pneumoniae* isolates with high-level carbapenem resistance.

**Method:**

A total of 80 *K. pneumoniae* isolates were purified from sputum and urine specimens. The minimum inhibitory concentrations (MICs) of imipenem and meropenem were determined by broth microdilution method. Carbapenemases were detected by Modified Hodge test and PCR. Additionally, the copy numbers of the identified genes (*bla*_VIM-1_, *bla*_NDM-1_ and *bla*_OXA-48_) were quantified by RT-PCR. The outer membrane proteins OmpK35 and OmpK36 of the resistant isolates were analyzed.

**Results:**

Eight isolates were resistant to carbapenems; six of these isolates possessed elevated MICs to imipenem and meropenem (≥16 µg/ml). Carbapenem resistant isolates harbored *bla*_NDM-1_ (n=5), *bla*_VIM-1_ (n=4) and *bla*_OXA-48_ (n=1) with some isolates had multiple carbapenemases genes. Six isolates with high MICs to imipenem contained multi-copies of the carbapenemases genes along with the lack of OmpK35. Isolates with intermediate resistance to carbapenems (MIC; 4-8 µg/ml) did not exhibit multiple carbapenemases but lacked the OmpK35. Random amplified polymorphic DNA exhibited three different patterns and indicated that five isolates encoded the same pattern P1.

**Conclusion:**

This study elucidated that multiple carbapenemases genes, high copy number of carbapenemases and loss of the porin OmpK35 could collectively contribute to the emergence of *K. pneumoniae* isolates with high resistance to carbapenems. Hence, more restrictions should be applied on the use of carbapenems to reduce the emergence of the resistant clones.

## INTRODUCTION

*K. pneumoniae* is a Gram-negative opportunistic pathogen associated with hospital-acquired infections such as pneumonia, urinary tract infections, septicemia and meningitis. Infection with multidrug-resistant *K. pneumoniae* isolates is a growing clinical problem [1]. Carbapenems are broad spectrum antibacterial agents that are kept as the last treatment option for infections caused by multi-drug resistant *K. pneumoniae* isolates [2]. However, uncontrolled administration of carbapenems leads to the development and the spread of carbapenem resistant isolates [3] which are usually resistant to other antibacterial agents such as fluoroquinolones [4], and these isolates have limited treatment options leading to significantly high morbidity and mortality rates [5].

Carbapenem resistance in *Enterobacteriaceae* has been developed due to the acquisition of genes encoding carbapenemases (carbapenem hydrolyzing enzymes) [3]. Three classes of carbapenemases (A, B and D) are involved in the carbapenem resistance i) class A (*K*. *pneumoniae* carbapenemase) [3, 4, 6, 7] ii) class B, metallo-β-lactamases (MBLs) such as Verona integron metallo-beta-lactamase (VIM), Imipenemase metallo-beta-lactamase (IMP) and New Delhi metallo-beta-lactamase (NDM) [5, 8] and iii) class D, oxacillin hydrolyzing beta-lactamases (OXA) [9, 10]. The carbapenem hydrolyzing genes are commonly encoded on mobile genetic elements and are accompanied by other antibiotic resistant genes resulting in cotransfer of the antimicrobial resistance genes and rapid spread of bacterial infections [5].

Furthermore, mutations in the bacterial outer membrane proteins contribute to the microbial resistance to carbapenems. For example, the loss of porins, OmpK35 and OmpK36, is associated with reduced susceptibility of *K. pneumoniae* to carbapenems and cephalosporins [11-14].

Treatment of life threatening infections with imipenem/meropenem can favor the development of resistance to carbapenem antibiotics. Recently, carbapenem-resistant *K. pneumoniae* strains emerged in Egyptian hospitals. In order to control the spread and dissemination of carbapenem-resistant *K. pneumoniae*, various mechanisms mediating the resistance of these isolates need to be investigated. In this research, different mechanisms behind the emergence of *K. pneumoniae* isolates with elevated resistance to carbapenems were clarified.

## METHODS

### Isolation and Purification of *K. Pneumoniae* Isolates

*K. pneumoniae* isolates were purified from urine and sputum samples in the period from January 2015 to June 2015 from Mansoura University hospitals according to the Helsinki declaration for medical research in handling, use and care of human subjects. *K. pneumoniae* isolates were identified in accordance with the biochemical standard assay methods [15]. The carbapenem susceptible *K. pneumoniae* ATCC 33495 was used as a control.

### Antibiotic Susceptibility Test of *K. Pneumoniae* Isolates

The susceptibility of all purified *K. pneumoniae* isolates to different carbapenems including imipenem (IPM, 10 µg), meropenem (MEM, 10 µg), doripenem (DOR, 10 µg) and ertapenem (ETP, 10 µg) was evaluated by the disc-diffusion method [16]. *K. pneumoniae* isolates with intermediate susceptibility to carbapenems and carbapenem resistant isolates were selected. Phenotypic and genotypic characterization of the carbapenem resistant isolates was conducted.

The selected carbapenem-resistant isolates were tested for susceptibility to other antimicrobial agents: (Table **2**).

### Minimum Inhibitory Concentrations of Imipenem and Meropenem

The minimum inhibitory concentrations for the carbapenem-resistant isolates against imipenem and meropenem were evaluated by broth microdilution method using 96-well microtiter plate according to the Clinical Laboratory Standards Institute (CLSI), 2015 [16].

### Phenotypic Screening of Carbapenemases Among the Carbapenem Resistant Isolates

The ability of *K. pneumoniae* isolates to release carbapenemases was estimated by Modified Hodge test (MHT) as recommended by CLSI, 2015 [16].

### PCR Analysis of the Carbapenem-Resistant Isolates

Total DNA was extracted from the carbapenem-resistant *K. pneumoniae* isolates using Genomic DNA purification Kit (Thermo Fisher Scientific, European Union). The extracted DNA was used in real time PCR and in random amplified polymorphic DNA (RAPD). The presence of genes coding for carbapenemases such as *bla*_VIM-1_, *bla*_NDM-1_, *bla*_IMP_, *bla*_KPC_ and *bla*_OXA-48_ was detected by polymerase chain reaction using fermentas Dream TaqTM Green PCR Master Mix and specific primer pairs (Table **1**) [6, 8, 9].

### Copy Number Analysis of the Carbapenem Resistance Genes

The copy numbers of the detected genes *bla*_VIM-1_, *bla*_NDM-1_ and *bla*_OXA-48_ were quantified by real-time polymerase chain reaction using FIREPol^®^ EvaGreen^®^qPCR Kit (Solis Bio-Dyne, Estonia). PCR consisted of 200 ng DNA, 1 μl of the forward primer (2 µM), 1 μl of the reverse primer (2 µM) (Table **1**), 4 μl of 5 × EvaGreen^®^qPCR master mix, and RNase-free water to a final volume of 20 μl. In addition, a negative control reaction lacking DNA template was performed. The quantitative PCR was processed in the Rotor-Gene Q thermocycler (QIAGEN, Germany) as indicated: one cycle at 95 °C for 15 min, then 40 repeated cycles of 15 seconds denaturation at 95 °C for, 30 seconds annealing at 55 °C with *bla*_OXA-48_ or 52 °C with *bla*_VIM-1_ and *bla*_NDM-1_ and 1 min extension at 72 °C. The copy numbers of the carbapenemase genes (*bla*_NDM-1,_ *bla*_OXA-48,_ *bla*_VIM-1_) were measured relative to an internal *K. pneumoniae* housekeeping gene *rpoD*. Standard curves were generated for both the target genes (*bla*_NDM-1,_ *bla*_OXA-48_ and *bla*_VIM-1_) and the endogenous control (*rpoD*) using 10-fold dilutions of the template DNA at known concentrations. In the standard curve, the logarithm of initial quantity of template was plotted against the *C_T_* values and gene copy numbers were calculated using the standard curves. The ratio of the target gene copy number to the reference gene copy number was calculated in order to determine the relative quantity of each gene [17, 18].

### Isolation and Characterization of the Outer-Membrane Proteins

The outer-membrane proteins of the carbapenem-resistant isolates were analyzed by SDS-PAGE [19] and compared to standard *K. pneumoniae* ATCC 33495. Briefly, the isolates were grown in nutrient broth, the cell pellets were sonicated and the cell debris was discarded. The supernatants were ultracentrifuged at 100.000 *×g* for one hour. The pellets were incubated with *N*-lauroylsarcosine (1 %) at 37 °C for 30 min. Again the outer membrane proteins were collected by ultracentrifugation at 100.000 *× g* for one hour. Samples were boiled in Laemmli's sample buffer. SDS-PAGE was performed with a 10 % acrylamide gel at 80 V for 3 hours and stained with Coomassie blue.

### Random Amplified Polymorphic DNA of the Carbapenem-Resistant Isolates

Molecular typing of the isolates was carried by random amplified polymorphic DNA (RAPD) using oligonucleotides Operon 18; ^5'^CAGCACCCAC^3'^ [20]. PCR mixture was composed of the genomic DNA (100 ng), Operon 18 primer (20 μM), 1 μl dNTP (250 μM each), 5× Flexi buffer GoTaq^®^, 1 μl MgCl_2_ (25mM), 1.5 U DNA polymerase FlexiTaq (Promega, USA) and H_2_O up to a final volume of 20 μl. The amplified amplicons were separated by electrophoresis on 1% w/v agarose gels at 80 V for 2 hours. The RAPD profile of the carbapenem-resistant isolates was scored as a binary code and analyzed using DendroUPGMA.

## RESULTS

### Antimicrobial Susceptibility and Carbapenem Resistance

In this study, eighty *K. pneumoniae* isolates were purified from urine (n=50) and sputum (n=30). Urine samples were collected from Kidney Hospital and Digestive System Center, and early morning sputum samples were collected from Oncology Hospital, Emergency Hospital, and Children Hospital. Eight isolates were resistant to doripenem and imipenem and seven isolates were resistant to ertapenem and meropenem. Table (**2**) demonstrates the epidemiological sources of the carbapenem resistant isolates along with the antimicrobial susceptibility profile against different antimicrobials. Four isolates were obtained from urine samples and four isolates were purified from early morning sputum. Antimicrobial susceptibility profiles of the carbapenem-resistant isolates against different antimicrobials agent are indicated in Table (**2**). The imipenem and doripenem non-susceptible isolates exhibited coresistant to other β-lactams; ampicillin/sulbactam, amoxicillin/clavulanic acid, ceftazidime, cefuroxime, ceftriaxone and cefotaxime. Also, seven of the aforementioned isolates were resistant to tobramycin and piperacillin/tazobactam. Furthermore, six isolates were resistant to cefoxitin, cefepime, gentamicin and ciprofloxacin. On contrast, most of the isolates retained susceptibility to trimethoprim-sulfamethoxazole (n=4), levofloxacin (n=6) and amikacin (n=6).

### Minimum Inhibitory Concentrations and Carbapenemases

Table (**3**) shows the minimum inhibitory concentrations for the carbapenem-resistant isolates against imipenem and meropenem. All the resistant isolates had high MICs (≥16 µg/ml) except isolates K18 and K70 had MICs from 4-8 µg/ml. The carbapenem-resistant isolates developed a distorted or a cloverleaf shaped inhibition zone in the MHT as a positive indication of carbapenemases. In addition, PCR showed that five isolates (K18, K23, K40, K70 and 74) harbored *bla*_NDM-1_ gene, four isolates harbored *bla*_VIM-1_ gene (K11, K23, K35 and K60), K23 had both *bla*_NDM-1_ and *bla*_VIM-1_ genes and K60 carried dual resistance genes *bla*_VIM-1_ and *bla*_OXA-48_ genes. PCR also showed that all the carbapenem-resistant isolates lacked *bla*_KPC_ and *bla*_IMP_ carbapenemases.

### Gene Copy Numbers and Elevated Resistance to Carbapenems

The replicates of carbapenemase encoding genes were detected by RT-PCR. Isolates K23, K40 and K74 had high copies of *bla*_NDM-1_. Also, multi-copies of *bla*_VIM-1_ were detected in isolates K11, K35 and K60. Meanwhile, isolate K60 recorded a high copy number for *bla*_OXA-48_. In contrast, isolates K18 and K70 had low copy numbers of *bla*_NDM-1_ (Table **3 **).

Data represented as the mean of two independent experiments **±** standard deviation.

### Porin Analysis

**Fig. (1):**
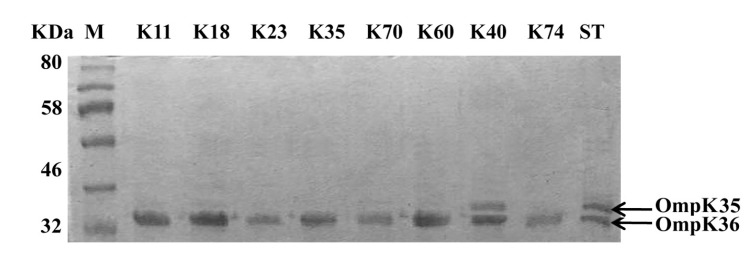
**Sodium dodecyl sulphate poly acrylamide gel electrophoresis (SDS-PAGE) of the outer membrane proteins OmpK35 and OmpK36 of the carbapenem resistant isolates compared to the standard *K. pneumoniae* ATCC33495.** M: prestained protein marker; ST: Standard *K. pneumoniae*.

The outer membrane proteins of the carbapenem-resistant isolates were prepared and separated on SDS-PAGE. All isolates had intact outer membrane protein OmpK36. However, OmpK35 could not be detected among all tested isolates except isolate K40 which contained both porins OmpK35 and OmpK36 as shown in (Fig. **1**).

### Random Amplified Polymorphic DNA

**Fig. (2):**
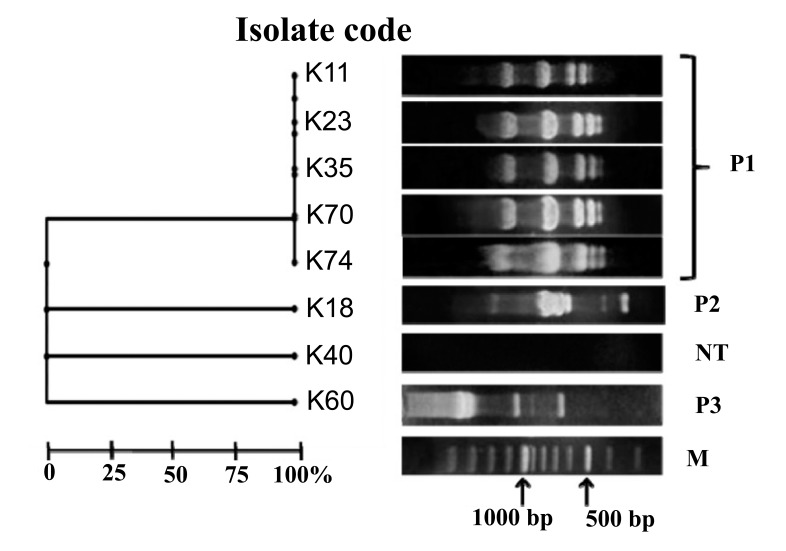
**Random Amplified Polymorphic DNA (RAPD) of *K. pneumoniae* isolates resistant to carbapenems**. They assigned RAPD types P1, P2 and P3. The scale indicates similarity percentage. M lane represents the DNA marker (100 base pair plus). NT: non typeable.

In the present study, carbapenem-resistant isolates were categorized as three RAPD patterns named P1, P2 and P3. Five *K. pneumoniae* isolates K11, K23, K35, K70 and K74 shared the same pattern P1. Patterns P2 and P3 were represented by isolates K18 and K60, respectively. However, isolate K40 was non typeable (Fig. ** 2**).

## DISCUSSION

Carbapenems have been kept as the last antibiotic choice for the treatment of severe infections, however, the development of carbapenem resistance significantly compromises their activities [2]. Carbapenem resistance is widely spread all over the world and is becoming worrisome [9]. Hence, characterization of the carbapenem-resistant isolates is the first step in the road map for controlling these isolates [4]. In this study, eight isolates were found to be resistant to carbapenems as well as various classes of antimicrobial agents (Table **2**). Similarly, carbapenem-resistant *K. pneumoniae* isolates exhibited coresistance to amoxicillin/clavulanic acid, ampicillin/sulbactam, cefuroxime and 3^rd^ generation cephalosporins [13, 21].

The carbapenem-resistant isolates were classified into two groups according to the level of susceptibility to both imipenem and meropenem. The first group included isolates K11, K23, K35, K40, K60 and K74 with high resistance level to imipenem and meropenem (MIC≥16 µg/ml). The second group involved isolates K18 and K70 that were less resistant to both carbapenems (MICs from 4- 8 µg/ml) (Table **3**).

The carbapenem-resistant isolates were carbapenemase positive and harbored the carbapenemase encoding genes; *bla*_NDM-1_, *bla*_VIM-1_ and *bla*_OXA-48_ particularly, isolate K23 and K60 had multiple carbapenemases genes. These results revealed that class B carbapenemase are predominant in *K. pneumoniae* isolates assessed in this study. Various studies detected carbapenemase genes in the carbapenem-resistant *K. pneumoniae* isolates especially *bla*_VIM_ and *bla*_NDM_ however, carbapenemase *bla*_IMP_ was not common in *Enterobacteriaceae* [9, 22, 23]. *K. pneumoniae* contained multiple metallo-β-lactamase genes have been previously detected in Germany [21], Italy [24], Colombia [25] and Sultanate of Oman [26].

Additionally, both isolates K23 (*bla*_NDM-1_/*bla*_VIM-1_) and K60 (*bla*_OXA-48_/*bla*_VIM-1_) carried high copy number of carbapenemase genes (Table ** 3**). Likewise, *K. pneumoniae* harbor multi-copies of *bla*_TEM_, *bla*_CTX-M_, and *bla*_SHV_ [27] which conveys high resistance to β-lactams. Furthermore, multi-copies of *bla*_NDM-1_ were carried on pKPX-1 plasmid in the form of tandem repeats which contributed to high-level resistance to carbapenems in *K. pneumoniae* isolate KPX [28]. On the other hand, both isolates K18 and K70 were less resistant to imipenem and meropenem, and contained low copy numbers of *bla*_NDM-1_. Hence, carbapenem resistance in these isolates could be attributed to other mechanisms. Loss of OmpK35 or OmpK36 porins has been associated with resistance to carbapenems and cephalosporins [13, 29]. Therefore, we examined the porins as an additional resistance mechanism. Kitchel, and coauthors [18] have detected that the lack of functional OmpK35 was prominent among 80% of *K. pneumoniae* isolates and only four isolates with low-level carbapenem resistance had mutation in OmpK36 sequences. On the same instance, our SDS-PAGE indicated intact OmpK36 in all carbapenem-resistant isolates and all isolates lost OmpK35 except isolate K40. Hence, the high carbapenem resistance could be attributed to multi-copies of carbapenemases and lack of OmpK35 (Fig. **1**) as combined mechanisms of carbapenem resistance. Intermediate level of resistance to carbapenems in isolates K18 and K70 may be due to the loss of the OmpK35. On the other hand, isolate K40 with high-resistance level contained intact porins, and its resistance pattern can be attributed to the high copy number of *bla*_NDM-1_ (Table **3**).

Epidemiological classification of the carbapenem resistant isolates assigned three patterns P1, P2 and P3. Pattern P1 was the most predominant one where it displayed the profile of five *K. pneumoniae* isolates with 100% similarity. In the P1 profile, three isolates contained *bla*_NDM-1_ (K23, K70 and K74) and three isolates harbored *bla*_VIM-1_ (K11, K23 and K35) Fig. (**2**). This illustrated a possible genetic similarity between isolates encoding both *bla*_VIM-1_ and *bla*_NDM-1_.

## CONCLUSION

In the present research, *K. pneumoniae* isolates were resistant to carbapenems and to several antibiotics. MHT and PCR confirmed the presence of carbapenemases and multiple-carbapenemases in the isolates. Moreover, this study highlighted the prevalence of multi-copies of *bla*_NDM-1_ and *bla*_VIM-1_ in *K. pneumoniae* isolates with high-resistance levels. Loss of OmpK35 also assisted in carbapenem resistance to certain extent. The situation is worrisome as the available treatment options are limited and the development of novel molecules to control this invasive infection is not easily accomplished. Hence, more restrictions on the administration of carbapenems should be applied in our hospitals to reduce the spread of the carbapenem-resistant *K. pneumoniae* isolates.

## Figures and Tables

**Table 1:** Primer pairs used in PCR and RT-PCR of carbapenemases.

**Gene type**	**Primer**	**Nucleotide sequence**		**Annealing Temp**	**Amplicon size (bp)**
**Carabapenemase genes internal primers**	**OXA-48**	**F**	**5`– AAGTGTGCAACGCAAATGGC – 3`**	**55°C**	**137**
		**R**	**5` – CTGTTCCAGATCTCCATTCC – 3`**		
	**IMP**	**F**	**5` – GTTAGTCACTTGGTTTGTG – 3`**	**50°C**	**103**
		**R**	**5` – CGAGAATTAAGCCACTCTA – 3`**		
	**KPC**	**F**	**5` – ATTCGCTAAACTCGAACAG – 3`**	**50°C**	**130**
		**R**	**5` – AAGAAAGCCCTTGAATGAG – 3`**		
	**VIM-1**	**F**	**5` – GAGCTCTTCTATCCTGGTG – 3`**	**52°C**	**103**
		**R**	**5` – CTTGACAACTCATGAACGG – 3`**		
	**NDM-1**	**F**	**5` – ACTTCCTATCTCGACATGC – 3`**	**52°C**	**133**
		**R**	**5` – TGATCCAGTTGAGGATCTG – 3`**		
**Carabapenemase genes full length primers**	**OXA-48**	**F**	**5` – TTGGTGGCATCGATTATCGG – 3`**	**55°C**	**743**
		**R**	**5` –GAGCACTTCTTTTGTGATGGC – 3`**		
	**VIM-1**	**F**	**5` – TGTTATGGAGCAGCAACGATG – 3`**	**56 °C**	**920**
		**R**	**5` – AAAGTCCCGCTCCAACGATT – 3`**		
	**NDM-1**	**F**	**5` – TGTTATGGAGCAGCAACGATG – 3`**	**62°C**	**795**
		**R**	**5` – AAAGTCCCGCTCCAACGATT – 3`**		
**Housekeeping gene**	**RpoD**	**F**	**5` – CGAACTGCTTGCCGACTT – 3`**	**56°C**	**131**
		**R**	**5` – GCGAGAGCCTCAAGGATAC – 3`**		

F: forward, R: reverse, bp: base pair

**Table 2:** Susceptibility of the carbapenem resistant isolates to various antimicrobial agents.

**Isolate** **code**	**Clinical** **source**	**Hospital**	**Antimicrobial susceptibility profile**																		
			**AMC**	**SAM**	**FOX**	**CXM**	**CTX**	**CRO**	**CAZ**	**TZP**	**FEB**	**CN**	**TOB**	**AK**	**SXT**	**CIP**	**LEV**	**IPM**	**MEM**	**DOR**	**ETP**
**K11**	Sputum	Emergency hospital	**R**	**R**	**S**	**R**	**R**	**R**	**R**	**R**	**R**	**R**	**R**	**S**	**R**	**R**	**S**	**R**	**R**	**R**	**R**
**K18**	Sputum	Emergency hospital	**R**	**R**	**R**	**R**	**R**	**R**	**R**	**R**	**R**	**R**	**R**	**S**	**R**	**R**	**S**	**R**	**S**	**R**	**R**
**K23**	Sputum	Children hospital	**R**	**R**	**R**	**R**	**R**	**R**	**R**	**R**	**S**	**S**	**R**	**S**	**S**	**R**	**R**	**R**	**R**	**R**	**R**
**K35**	Urine	Kidney center	**R**	**R**	**R**	**R**	**R**	**R**	**R**	**R**	**S**	**R**	**R**	**S**	**S**	**S**	**S**	**R**	**R**	**R**	**R**
**K40**	Urine	Digestive system center	**R**	**R**	**R**	**R**	**R**	**R**	**R**	**R**	**R**	**R**	**R**	**R**	**R**	**S**	**S**	**R**	**R**	**R**	**R**
**K60**	Sputum	Children hospital	**R**	**R**	**R**	**R**	**R**	**R**	**R**	**R**	**R**	**R**	**R**	**S**	**R**	**R**	**S**	**R**	**R**	**R**	**R**
**K70**	Urine	Kidney center	**R**	**R**	**S**	**R**	**R**	**R**	**R**	**I**	**R**	**S**	**S**	**S**	**S**	**R**	**S**	**R**	**R**	**R**	**I**
**K74**	Urine	Kidney center	**R**	**R**	**R**	**R**	**R**	**R**	**R**	**R**	**R**	**R**	**R**	**R**	**S**	**R**	**R**	**R**	**R**	**R**	**R**

AMC: amoxicillin/clavulanic acid, SAM: ampicillin/sulbactam, FOX: cefoxitin, CXM: cefuroxime, CTX: cefotaxime, CRO: ceftriaxone, CAZ: ceftazidime, TZP: piperacillin-tazobactam, FEP: cefepime, CN: gentamicin, TOB: tobramycin, AK: amikacin, SXT: trimethoprim-sulfamethoxazole, CIP: ciprofloxacin, LEV: levofloxacin, IPM: imipenem, MEM: meropenem, DOR: doripenem, ETP: ertapenem.

**Table 3:** Characteristics of the carbapenem resistant isolates.

**Isolate code**	**MIC (μg/ml)**		**PCR of** **β-lactamases/carabapenemases**					**Relative Copy number**		
	**IPM**	**MEM**	***bla*** **_IMP_**	***bla*** **_KPC_**	***bla*_OXA-48_**	***bla*** **_NDM-1_**	***bla*** **_VIM-1_**	***bla*_OXA-48_**	***bla*_NDM-1_**	***bla*_VIM-1_**
**K11**	**256**	**128**	**-**	**-**	**-**	**-**	**+**	**ND**	**ND**	**5.25±0.30**
**K18**	**4**	**0.5**	**-**	**-**	**-**	**+**	**-**	**ND**	**0.24±0.01**	**ND**
**K23**	**1024**	**512**	**-**	**-**	**-**	**+**	**+**	**ND**	**11.61±2.89**	**0.21±0.17**
**K35**	**64**	**32**	**-**	**-**	**-**	**-**	**+**	**ND**	**ND**	**6.67±1.31**
**K40**	**128**	**128**	**-**	**-**	**-**	**+**	**-**	**ND**	**10.70±2.49**	**ND**
**K60**	**128**	**512**	**-**	**-**	**+**	**-**	**+**	**13.17±1.32**	**ND**	**2.44±0.13**
**K70**	**8**	**4**	**-**	**-**	**-**	**+**	**-**	**ND**	**0.16±0.01**	**ND**
**K74**	**16**	**16**	**-**	**-**	**-**	**+**	**-**	**ND**	**3.13±0.50**	**ND**

IPM; imipenem, MEM: meropenem, ND: non-detectable, MIC: minimal inhibitory concentration.

## LIST OF ABBREVIATIONS

MIC: = Minimum inhibitory concentrations
RAPD: = Random amplified polymorphic DNA
MHT: = Modified Hodge test
MBLs: = Metallo-β-lactamases
RT-PCR: = Real-time polymerase chain reaction
